# RELATED FACILITY AND RESIDENT FACTORS ON STANDARDIZED NURSING LANGUAGES FOR NURSING HOME NURSES IN KOREA

**DOI:** 10.1093/geroni/igae098.2801

**Published:** 2024-12-31

**Authors:** Juh Hyun shin, Chung Hyuk Park, Myungeun Lee, Suhyun Park

**Affiliations:** George Washington University, Ashburn, Virginia, United States; George Washington University, Washington, District of Columbia, United States; George Washington University, Washington, District of Columbia, United States; University of Minnesota, Minneapolis, Minnesota, United States

## Abstract

Standardized nursing languages play a crucial role in facilitating holistic nursing care, but their full benefits are still being realized. However, the implementation of NNN linkages and their impact on nursing processes and nursing home (NH) residents’ outcomes, as well as the identification of facility and resident factors, remain unexplored. The purpose of this study was to examine (a) frequently occurring NNN linkages and (b) related facility and resident factors on the use of NANDA, NIC, and NOC for NH residents. Data were collected from 273 residents in 19 NHs in Korea 19 NHs in Korea, with structured questionnaires administered to NH registered nurses using a newly developed smartphone application. Descriptive statistics, ANOVA, and ANCOVA were used for the data analysis. We identified that factors, such as case mix index (resident acuity), age of resident, year of facility establishment, facility ownership status, and admission period of residents, are related to the use of NANDA, NIC, and NOC. Nine NNN linkages were newly identified in this study, mostly centered on fall prevention. The findings provide various approaches to address the risk of falls in NHs considering environmental, physical, and psychological factors. This study contributes to the foundation of evidence related to NHs by collecting specific data on the nursing process for the physiological, behavioral, safety, family, health system, and community areas applied to the residents of long-term care facilities at the national level. Future research in different settings and tailored population is needed.

